# Effects of natamycin and *Lactobacillus plantarum* on the chemical composition, microbial community, and aerobic stability of *Hybrid pennisetum* at different temperatures

**DOI:** 10.1039/d0ra00028k

**Published:** 2020-02-27

**Authors:** Assar Ali Shah, Chen Qian, Juanzi Wu, Zhiwei Liu, Salman Khan, Zhujun Tao, Xiaomin Zhang, Irfan Ullah Khan, Xiaoxian Zhong

**Affiliations:** National Forage Breeding Innovation Base (JAAS) Nanjing 210014 P. R. China zhpansy@aliyun.com xiaoxian@jaas.ac.cn +86-25-84390345 +86-25-84390239; Institute of Animal Science, Jiangsu Academy of Agricultural Sciences Nanjing 210014 P. R. China; Key Laboratory for Crop and Animal Integrated Farming, Ministry of Agriculture and Rural Affairs Nanjing 210014 P. R. China; School of Life Sciences, State Key Laboratory of Pharmaceutical Biotechnology, Nanjing University Nanjing 210023 P. R. China; Department of Biochemistry and Molecular Biology, College of Life Science, Nanjing University Weigang, No. 1 Nanjing 210095 P. R. China

## Abstract

This study evaluated the effects of natamycin and *Lactobacillus plantarum* on the chemical composition, microbial community, and aerobic stability of *Hybrid pennisetum* at different temperatures. Different concentrations of natamycin (0.50 g L^−1^, 1.00 g L^−1^, and 1.50 mg L^−1^) significantly (*p* > 0.05) reduced the growth of undesirable microorganisms. During the ensiling periods the pH, ammonia nitrogen (NH_3_–N), acetic acid (AA), butyric acid (BA), aerobic bacteria (AB), and yeast were significantly (*p* > 0.05) reduced, while the lactic acid and lactic acid bacteria were significantly (*p* < 0.05) influenced in the SLP and SLNP groups as compared to the SP and SNP groups at high temperature (29–30 °C). During air exposure, water-soluble carbohydrate, ammonia nitrogen (NH_3_–N), lactic acid (LA), and acetic acid (AA) were not influenced, while pH and aerobic bacteria were significantly (*p* < 0.05) enhanced after three days (72 hours) of air exposure, and lactic acid bacteria were significantly (*p* > 0.05) reduced at ambient temperature (9–10 °C). It is concluded that the addition of *L. plantarum* CICC 20765 alone and in combination with natamycin reduced the content of AA, pH, NH_3_–N, BA, and undesirable microbial community, and enhanced the chemical composition, fermentation quality, and air exposure. Natamycin alone did not significantly enhance the organic acid profile but improved the air exposure. Furthermore, more effort is needed to evaluate the effects on silage preservation on a large scale and on animal performance.

## Abstract 2

The inoculation of *L. plantarum* and natamycin influenced the fermentation quality. Natamycin and *L. plantarum* reduced the undesirable microbial community. During ensiling process, the LA and LABs was significantly enhanced.

## Graphical abstract

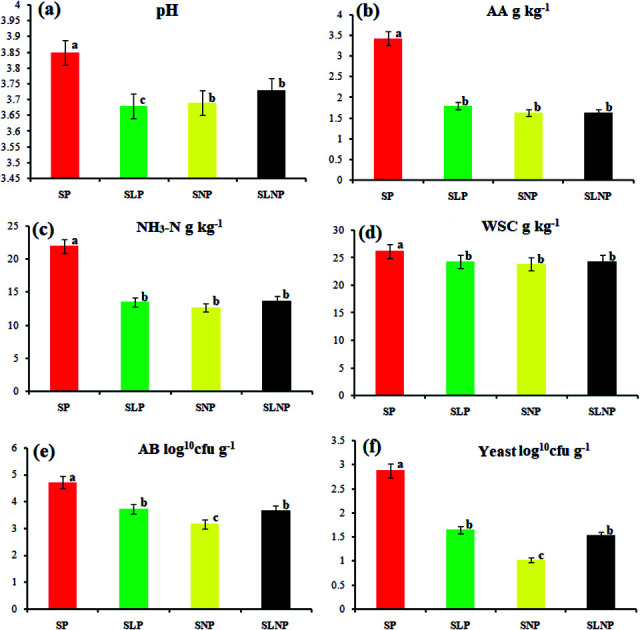

## 1 Introduction

Inoculation with antimicrobials is mostly used to inhibit the growth of yeasts, fungi, and other undesirable microorganisms to achieve better fermentation quality and improve the aerobic stability of *Hybrid pennisetum* (Tift 23A × Sumu No. 2 Napier grass) silages. *Hybrid pennisetum* is commonly used as a bio-energy grass and might also serve as forage for livestock production. *Hybrid pennisetum* is a major tropical grass and one of the highest yielding tropical grasses. It is a very resourceful species that can be developed under a wide range of environmental conditions, such as wet or dry environments, by smallholders or for larger-scale agriculture. It is an important forage and is very popular throughout the tropics and sub-tropics, especially in cut-and-carry systems.^1^

Natamycin is occasionally called pimaricin. Natamycin is a polyene macrolide antibiotic that is active against yeasts and molds. It is commonly used as a food stabilizer all over the world.^2,3^ Recently, natamycin has been used worldwide as a preservative of human and animal feed, for example, meats, dairy products, fruits, and vegetables.^4^ Natamycin reduced the growth of *Aspergillus* spp., *Cephalosporium* spp., *Candida* spp., *Fusarium* spp. and *Penicillium* spp., as well as yeasts and fungi, but is inactive against Gram-positive and Gram-negative aerobic and anaerobic bacteria.^3^ The growth of fungi, molds, and yeast mostly enhance during ensiling and after silage opening. These undesirable microorganisms produce ammonia N, affecting the silage quality, which means lactic acid bacteria (LAB) fail to create adequate lactic acid during the fermentation to reduce the pH and inhibit the growth of *Clostridia*.^4,5^ In the present study, natamycin is used to reduce the growth of fungi, molds, and yeast during and after ensiling processes.^2,4^

Natamycin is beneficial for animal and human disease treatment and the protection of foods and feeds. Natamycin is recommended over many other preservative products because it is free from odor and color so it does not cause taste aversion in feed or food.^4^ So there are no published reports about grass fermentation or grass preservation. Therefore, the aim of the current study was to investigate the effects of natamycin on silage fermentation quality and the effects of different concentrations effects of natamycin on the growth of lactic acid bacteria (LAB), yeasts, molds, and enterobacteria.

Ensiling is a fermentation process in which LAB and temperature both play important roles. LAB have a wide range of growth temperatures (5–50 °C) and different LAB have different optimal growth temperatures.^5,6^ In addition, during the initial period of ensiling, when air is still present between the plant particles and the silo, the temperature might rise to 40 °C or above because of continuing plant respiration and aerobic microbial activity.^7,8^ Many researchers have focused on improving aerobic stability by inoculating herbage with LAB because they produce antifungal substances during ensiling.^9–11^ Moreover, the temperature may influence the yield of antifungal substances produced by the LAB. Prema *et al.*^12^ reported that *L. plantarum* MiLAB393 had higher 3-phenyllactic acid production at 30 °C than at other temperatures. *L. buchneri* PW01 showed lower anaerobic conversion from lactic acid to acetic acid at 15 °C than 20 °C, 25 °C and 30 °C. Thus, it is concluded that the LAC species have a significant effect on feed perseveration and aerobic stability.^13^ Accordingly, Shah *et al.*^6^ reported that the addition of *L. plantarum* spp. (commercial and isolate) enhanced the feed preservation characteristic and aerobic stability of king grass silage.

The addition of anti-microbial and chemical agents is commonly used to stop the development of yeast, fungi and other undesirable microbes in order to improve the fermentation quality and aerobic stability of the silage.^10,13^ There are no reviews or literature about the effects of the addition of natamycin on silage fermentation quality and aerobic stability. Therefore, the aim of the current study was to investigate the effects of natamycin and *Lactobacillus plantarum* on the chemical composition, microbial community, and aerobic stability of *Hybrid pennisetum* at different temperatures.

## 2 Materials and methods

### 2.1 Field site

'The laboratory experiment was conducted in Prof. Xiaoxian Zhong's lab at the Institute of Animal Sciences, Jiangsu Academy of Agricultural Sciences, Nanjing, and the field site experiment was conducted at Jinci Village Zhuzhen Town, Liuhe District Nanjing, P. R China. *Hybrid pennisetum* was planted on 8 June 2018 (planted in summer season), in an experimental field (humid subtropical climate, latitude 32°01′ 59.81′′N, longitude 118°50′ 13.63′′E, and altitude of 17 m above mean sea level). *Hybrid pennisetum* was harvested at the mature stage on 1st December 2018 (at the start of winter).

### 2.2 Effects of different concentrations of natamycin on microbial community growth

Before the ensiling, 20 g of fresh *Hybrid pennisetum* was placed in a conical flask with 180 mL sterilized water (NaCl 8.50 g L^−1^) and shaken using a medium-speed auto shaker incubator (Crystal, 1S-RDV1) for 2 hours. After 2 hours, 100 μL of the silage extract was taken from the conical flask and added to a tube, to make four serial dilutions using NaCl (8.50 g L^−1^) sterilized water. The solutions were mixed well and 50 μL of the silage extract was spread on agar media plates with different concentrations of natamycin. In addition, the different concentrations effects of natamycin on some isolated and natural microbes, such as LAB, yeast, anaerobic bacteria and enterobacteria, were determined. Natamycin was added to four kinds of agar media at different concentrations, such as a control (0.00 mg L^−1^), 10.00 mg L^−1^, 20.00 mg L^−1^, 0.50 g L^−1^, 1.00 g L^−1^, and 1.50 g L^−1^. 50 μL aliquots of LAB were spread on Rogosa and Sharp (MRS) (Hopebio, Qingdao Hope Bio-Technology Co., Ltd.) agar plates and placed in an anaerobic incubator at 37 °C for 3 days. The enterobacteria were spread on Violet Red Bile agar plates, yeasts were spread on nutrient agar (NA) plates and anaerobic bacteria were spread on potato dextrose agar (PDA) plates and placed in an aerobic incubator at 37 °C for 3 days. After 3 days, the microbial population data were recorded and the data were transformed to log^10^ and analyzed the microbial data.

### 2.3 Preparation of the laboratory silages

Fresh *Hybrid pennisetum* was chopped into 2–3 cm long pieces using a stationary electrical chopping machine (Sh-2000, Shanghai Donxe Industrial Co., Ltd., China) and ensiled in plastic laboratory silos (plastic drum, 1 L capacity, diameter 9.5 cm, height 18.7 cm, Lantian Biological Experimental Instrument Co., Ltd, Jiangsu, China). Each silo contained 700 g of fresh *Hybrid pennisetum* treated with the following four treatments: (1) control (SP), (2) *Lactobacillus plantarum* (CICC 20765, Ecosyl Products Inc. Chinese commercial bacteria adjusted at the rate of 1 × 10^5^ cfu g^−1^) (SLP), (3) natamycin (pimaricin containing 95% active compound) supplemented with 0.5 g kg^−1^ DM fresh weight (FW) of *Hybrid pennisetum* (SNP), and (4) the combination of the SLP and SNP treatments (SLNP). After treating and mixing, 10 mini-silos were prepared per treatment and packed into a plastic laboratory silo, followed by sealing with a screw top (internal and external), and all the mini-silos were kept at a high temperature (29–30 °C). The screw top was fitted with a synthetic fermentation trap filled with water to prevent the entry of air. Each mini-silo for each treatment was opened on days 5, 7, 14, 30, and 60.

### 2.4 Collection of samples for chemical and fermentative characteristics analyses

The king grass silages samples were collected at the 5th, 7th, 14th, 30th and 60th days. 80 g samples of *Hybrid pennisetum* fresh material and silage were taken for the determination of the dry matter (DM) content, which was measured by drying the samples in a forced-air oven at 65 °C for 72 hours and then grinding using a high-speed universal grinder (Hainai ship Hi-100C, Hainai Yinjiang Litong Trade Company Ltd., Zhejiang, China). The ground materials were used for neutral detergent fiber (NDF), acid detergent fiber (ADF), and water-soluble carbohydrate (WSC) content estimation *via* the anthrone–sulfuric acid method.^14,15^ A 35 g subsample of *Hybrid pennisetum* silage was macerated with 70 mL of distilled water and stored in a refrigerator at 4 °C for 24 hours. After 24 hours, the silage extract was filtered through double-layered cheesecloth and filter paper (Xinhua Co., China), and the pH of the silage extract was recorded using a pH meter (HANNA pH 211, Hanna Instruments Italia Sel, Italy). The filtered solution was stored at −20 °C for further analysis of ammonia nitrogen (NH_3_–N), and organic acid. Ammonia N was determined using the phenol–hypochlorite procedure.^15^

### 2.5 Organic acid analyses

A solution of metaphosphoric acid (HPO_4_) was prepared by using 100 ml sterilizing water dH_2_O for 2–3 hours shaker, because the HPO_4_ took a long time to dissolve it. After dissolving, the solution was made up to 100 mL before use. Next, 1.6 mL of silage extract was mixed well with 0.4 mL of HPO_4_ and kept at −20 °C for one night. The subsamples were then centrifuged at 13 000 rpm for 10 minutes at 16 °C. After the centrifuging, 1 mL of supernatant fluid was removed using a syringe and placed into HPLC vials by filtering through a 0.22 μm syringe filter (Shanyu Technology Development Co., Ltd.). After this protocol, the samples were analyzed by HPLC. An Agilent 1260 HPLC system (Agilent Technologies, Inc., Waldbronn, Germany) with a refractive index detector was used for the organic acid quantification with the following conditions: column: Carbomix® H-NP5, Sepax Technologies, Inc., Newark, DE, USA; eluent: 2.5 mmol L^−1^ H_2_SO_4_; flow rate: 0.5 mL min^−1^; temperature: 55 °C.

### 2.6 Air exposure test

After 60 days, the silos were opened and kept at low temperature (9–10 °C) for 4 days. Every 24 hours (or after one night) samples were taken for the determination of pH, lactic acid (LA), acetic acid (AA), propionic acid (PA), ammonia nitrogen (NH_3_–N), water-soluble carbohydrate (WSC), lactic acid bacteria (LAB), aerobic bacteria (AB) and yeast.

### 2.7 Analysis of microbial population during and after ensiling

The same protocol was used for microbial population analysis during and after ensiling. A 20 g silage subsample was taken in a conical flask and 180 mL of sterilized water (NaCl 8.50 g L^−1^) was added. The subsample was then incubated using a medium-speed auto shaker incubator (Crystal, 1S-RDV1) at 37 °C for 2 hours.^7,8,12^ After 2 hours, made a five-tube (each tube contains 900 μL) serial dilution from a NaCl (8.50 g L^−1^) sterilized water and takes a 100 μL extract from a conical flask and adds in a tube, to make 1 mL, and mixed it very well. After preparing all the tubes, 50 μL of LAB was spread on Rogosa and Sharp (MRS) (Hopebio, Qingdao Hope Bio-Technology Co., Ltd.) agar plates and placed in an anaerobic incubator at 37 °C for 3 days. The enterobacteria were spread on Violet Red Bile agar plates, yeasts were spread on nutrient agar (NA) plates and anaerobic bacteria were spread on potato dextrose agar (PDA) plates and placed in an aerobic incubator at 37 °C for 3 days. After 3 days of incubation, the microbial population data were recorded and transformed to log^10^ and analyzed the microbial data.

### 2.8 Statistical analysis

The data were analyzed using the GLM procedure (SPSS, version 12.0). The means of the significantly affected traits were separated using Duncan's multiple range test.^16^ A *P* value of less than 0.05 was considered to be statistically significant.

## 3 Results

**Fig. 1:**
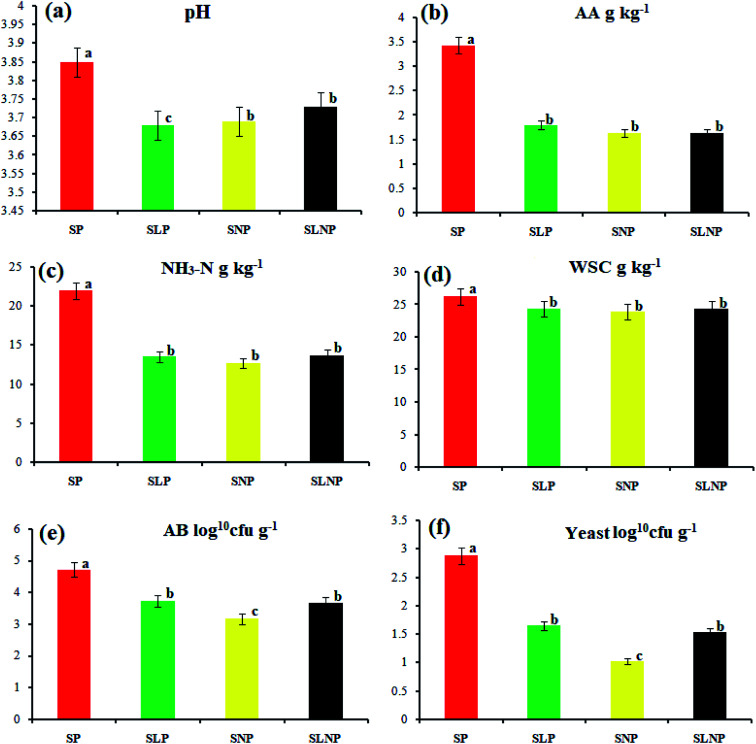
The *Hybrid pennisetum* fermentation quality after 60 days of ensiling.

**Fig. 2:**
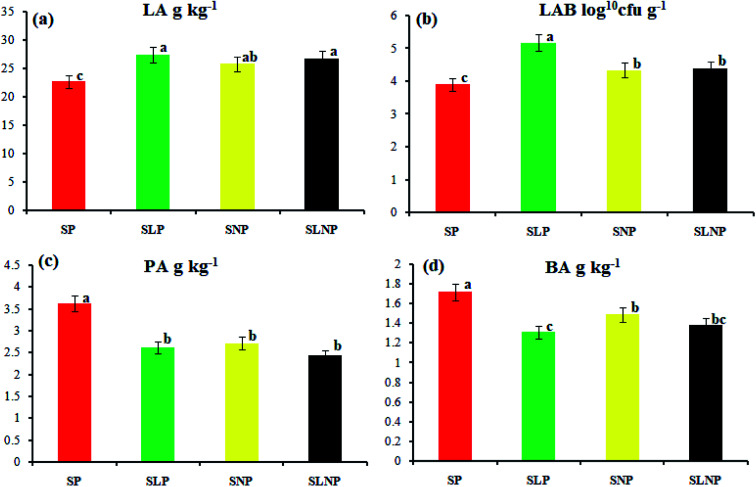
The change in organic acid content in *Hybrid pennisetum* silage at the 60th day of ensiling.

**Fig. 3:**
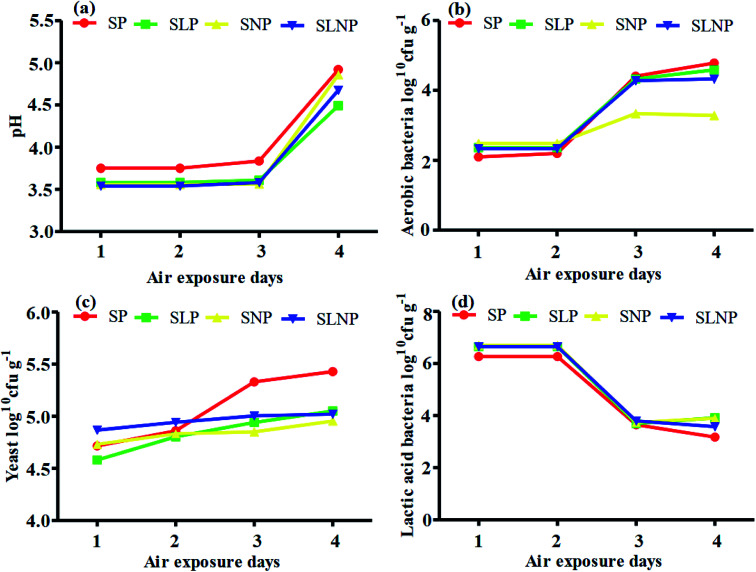
Changes in the fermentation quality and organic acid content of *Hybrid pennisetum* silage during air exposure.

**Fig. 4:**
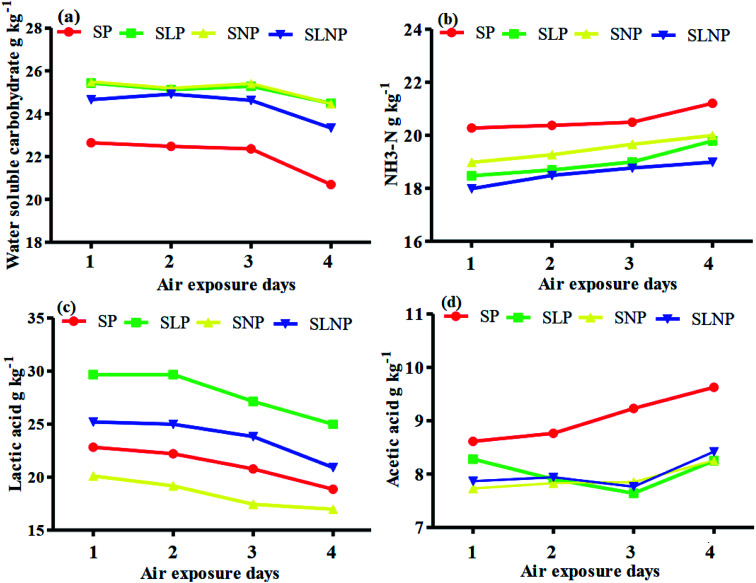
Changes in the microbial population in *Hybrid pennisetum* silage during air exposure.

The effects of different concentrations of natamycin on microbe growth in fresh grass and isolated microbe growth are illustrated in Table 1. The different concentrations of natamycin had different effects on the microbial growth, but the 0.50 g L^−1^, 1.00 g L^−1^, and 1.50 g L^−1^ concentrations significantly (*p* < 0.05) reduced the growth of yeast, aerobic bacteria, and enterobacteria, while lactic acid bacteria were not affected. The population dynamics of the three most important microbial groups of *Hybrid pennisetum* are illustrated in Fig. 1e, d, 2b, and Table 2. The interaction between different treatments (*T*) and different ensiling days (*D*), the aerobic bacteria (AB) and yeast were not affected at days 5 and 7, but at days 14, 30 and 60 they were significantly (*p* > 0.05) reduced, while LAB was significantly (*p* < 0.05) changed in all ensiling days and treatment groups, but the greatest significant (*p* < 0.05) differences were seen in the SLP and SLNP groups at high temperature (29–30 °C). The chemical composition and fermentation characteristics of *Hybrid pennisetum* before and after ensiling are illustrated in Tables 3–6. The interaction between different treatments (*T*) and different ensiling days (*D*), at days 5, 7, 14, 30, and 60 the crude protein (CP), dry matter (DM), neutral detergent fiber (NDF), and acid detergent fiber (ADF) were not affected while the ammonia nitrogen (NH_3_–N) and water-soluble carbohydrates (WSC) were significantly (*p* < 0.05) reduced in all treatment groups as compared to the control at high temperature (29–30 °C). The chemical compositions and fermentation characteristics of *Hybrid pennisetum* are illustrated in Fig. 1a, b, f and 2c, d, Tables 4 and 5. The interaction between different treatments (*T*) and different ensiling days (*D*), at days 5, 7, 14, 30, and 60, the pH, acetic acid (AA), butyric acid (BA) yeast were changed in all treatment groups but the greatest significant (*p* > 0.05) reductions were seen in the SLP and SLNP groups at high temperature (29–30 °C). The interaction between different treatments (*T*) and different ensiling days (*D*), at days 5, 7, and 14, the propionic acid (PA) was significantly (*p* < 0.05) increased but at days 30 and 60 it was not influenced while the lactic acid (LA) was significantly (*p* < 0.05) influenced in all treatment groups but the greatest significant (*p* > 0.05) influence was observed in the SLP and SLNP groups at high temperature (29–30 °C). The chemical compositions and fermentation characteristics of *Hybrid pennisetum* during the air exposure are illustrated in Fig. 3a–d and 4a–d. The water-soluble carbohydrate, ammonia nitrogen (NH_3_–N), lactic acid (LA), and acetic acid (AA) were not influenced, while the pH, and aerobic bacteria were significantly (*p* < 0.05) increased after 3 days (72 hours) and the lactic acid bacteria were significantly (*p* > 0.05) decreased at ambient temperature (9–10 °C).

**Table 1:** Effects of natamycin concentration on microbe growth in fresh grass and isolated microbes^a^

Natamycin concentration	Fresh grass microbes				Isolated microbes		
	LAB	Yeast	AB	Enterobacteria	LAB	Yeast	AB
Control	5.60^a^	5.52^a^	5.57^a^	5.52^a^	5.98^a^	5.87^a^	5.87^c^
10 mg L^−1^	5.60^a^	4.88^a^	5.30^a^	5.29^c^	5.98^a^	5.82^a^	5.73^c^
20 mg L^−1^	5.59^a^	3.64^b^	4.85^b^	4.17^b^	5.98^a^	5.52^a^	5.40^c^
0.5 g L^−1^	5.59^a^	3.34^b^	3.85^b^	3.39^c^	5.98^a^	4.00^b^	4.76^c^
1.00 g L^−1^	5.58^a^	3.01^c^	3.22^c^	1.76^c^	5.98^a^	2.63^b^	3.47^c^
1.5 g L^−1^	5.60^a^	0.92^c^	1.54^c^	ND^c^	5.98^a^	1.99^c^	2.20^c^
Std. error	0.039	0.334	0.442	0.130	0.042	0.231	0.345
Significant	0.915	0.000	0.000	0.000	0.857	0.000	0.000

a a, b, c, values with different lower case letters show significant (*p* < 0.05) differences among ensiling days in the same treatment.

**Table 2:** Microbial composition of *Hybrid pennisetum* grass during ensiling^a^

Item	Ensiling day	SP	SLP	SNP	SLNP	Significant			
						SEM	*T*	*D*	*T* × *D*
Lactic acid bacteria (log^10^ cfu g^−1^)	5	4.85^bA^	5.27^aA^	5.21^aA^	5.31^aA^	0.224	0.002	<0.001	0.03
	7	4.80^bA^	5.34^aA^	5.06^aA^	5.20^aA^				
	14	4.34^bAB^	5.50^aAB^	4.83^abAB^	4.65^abAB^				
	30	4.52^bAB^	5.27^Aab^	4.79^abAB^	5.23^aAB^				
Aerobic bacteria (log^10^ cfu g^−1^)	5	4.87^A^	4.63^A^	4.92^A^	4.21^A^	0.212	0.000	<0.001	0.05
	7	4.74^A^	4.55^A^	4.14^A^	4.55^A^				
	14	3.90^aB^	2.44^bB^	2.17^abB^	2.91^bB^				
	30	4.63^aB^	3.89^bB^	2.00^bB^	3.62^bB^				
Yeast (log^10^ cfu g^−1^)	5	4.49^A^	4.35^A^	4.56^A^	4.76^A^	0.433	0.000	<0.001	0.04
	7	4.72^A^	4.00^A^	4.27^A^	4.66^A^				
	14	4.06^aB^	2.81^bB^	1.40^bB^	2.63^abB^				
	30	4.28^aB^	1.65^bB^	1.02^bB^	3.53^bB^				

a a, b, c, values with different lower case letters show significant (*p* < 0.05) differences among ensiling days in the same treatment. SP: control; SLP: *Lactobacillus plantarum*; SNP: natamycin; SLNP: *Lactobacillus plantarum* + natamycin.

**Table 3:** Chemical composition of *Hybrid pennisetum* before ensiling

Item	*Hybrid pennisetum*
Dry matter (g kg^−1^)	308.95 ± 0.09
Buffering capacity (meq. kg^−1^ DM)	281.35 ± 0.10
Acid detergent fiber (g kg^−1^ DM)	349.19 ± 0.10
Neutral detergent fiber (g kg^−1^ DM)	663.37 ± 0.05
Water-soluble carbohydrate (g kg^−1^ DM)	45.93 ± 1.00
Lactic acid bacteria log^10^ cfu g^−1^)	5.60 ± 0.08
Aerobic bacteria (log^10^ cfu g^−1^)	4.97 ± 0.08
Yeast (log^10^ cfu g^−1^)	4.93 ± 1.11

**Table 4:** Chemical composition of *Hybrid pennisetum* during ensiling^a^

Item	Ensiling day	SP	SLP	SNP	SLN	Significant			
						SEM	*T*	*D*	*T* × *D*
DM (g kg^−1^)	5	309.9^A^	301.86^A^	30.19^A^	30.08^A^	3.111	0.466	0.213	0.08
	7	304.3^A^	308.47^A^	309.66^A^	308.41^A^				
	14	307.7^A^	311.49^A^	308.00^A^	312.21^A^				
	30	305.4^A^	309.91^A^	310.17^A^	310.40^A^				
NH_3_–N (g kg^−1^ of DM)	5	25.81^aAB^	14.89^bAB^	26.81^aAB^	15.35^bAB^	0.862	<0.000	0.052	0.041
	7	31.99^aAB^	18.14^bAB^	25.62^abAB^	17.32^bAB^				
	14	38.86^aAB^	18.97^bAB^	27.11^abAB^	18.99^bAB^				
	30	38.13^aA^	21.54^bA^	25.28^abA^	21.69^bA^				
Water-soluble carbohydrates (g kg^−1^ of DM)	5	14.56^cB^	23.94^aB^	20.59^bB^	13.86^cB^	0.848	0.019	0.001	0.051
	7	12.20^bB^	21.40^aB^	15.24^bB^	17.83^abB^				
	14	12.21^bC^	12.04^bC^	11.83^bC^	11.16^bC^				
	30	10.58^bBC^	15.41^bBC^	10.97^bBC^	22.80^aBC^				

a a, b, c, values with different lower case letters show significant (*p* < 0.05) differences among ensiling days in the same treatment. SP: control; SLP: *Lactobacillus plantarum*; SNP: natamycin; SLNP: *Lactobacillus plantarum* + natamycin.

**Table 5:** Fermentation characteristics of *Hybrid pennisetum* during ensiling^a^

Item	Ensiling day	SP	SLP	SNP	SLNP	Significant			
						SEM	*T*	*D*	*T* × *D*
pH	5	4.46^aA^	3.83^cA^	4.05^bA^	3.87^Ca^	0.015	0.012	<0.001	0.005
	7	4.42^aAB^	3.77^bAB^	3.89^bAB^	3.80^bAB^				
	14	3.83^aB^	3.34^bC^	3.72^aC^	3.72^aC^				
	30	3.91^aBC^	3.10^bBC^	3.75^bBC^	3.72^bBC^				
Lactic acid (g kg^−1^ of DM)	5	7.61^cC^	19.29^aC^	11.52^bC^	16.24^abC^	0.606	<0.000	<0.001	0.061
	7	8.70^cB^	22.00^aB^	13.50^bB^	20.09^aB^				
	14	13.70^bAB^	20.75^aAB^	18.93^aAB^	20.52^aAB^				
	30	20.42^bAB^	25.29^aAB^	23.73^abAB^	25.63^aAB^				
Acetic acid (g kg^−1^ of DM)	5	3.08^aC^	1.43^bC^	1.43^bC^	1.14^bC^	0.081	<0.000	0.04	0.002
	7	3.38^aBC^	1.82^bBC^	2.15^bBC^	1.75^bBC^				
	14	3.53^aBC^	1.83^bBC^	2.70^abBC^	1.89^aBC^				
	30	4.09^aA^	2.54^bA^	2.22^bA^	2.31^bA^				
Propionic acid (g kg^−1^ of DM)	5	4.17^bB^	4.92^aB^	3.66^bB^	4.88^aB^	0.401	0.007	0.002	0.001
	7	2.79^bAB^	6.14^aAB^	3.73^bAB^	6.85^aAB^				
	14	3.23^bAB^	6.35^aAB^	4.23^bAB^	8.04^Aab^				
	30	5.12	6.64	5.17	6.20				
Butyric acid (g kg^−1^of DM)	5	4.79^aAB^	0.35^bAB^	2.58^bAB^	1.94^bAB^	0.044	<0.000	0.013	0.06
	7	5.07^aAB^	0.61^bAB^	3.65^b^	3.33^abAB^				
	14	5.01^aAB^	1.84^bAB^	3.79^bAB^	4.01^bAB^				
	30	7.86^aA^	2.38^bA^	2.86^bA^	4.43^abA^				

a a, b, c, values with different lower case letters show significant (*p* < 0.05) differences among ensiling days in the same treatment. SP: control; SLP: *Lactobacillus plantarum*; SNP: natamycin; SLNP: *Lactobacillus plantarum* + natamycin.

**Table 6:** Chemical compositions and fermentation characteristics of *Hybrid pennisetum* at 60th day of ensiling^a^

Item	Ensiling day	Groups				SEM	Significant
		SP	SLP	SNP	SLNP		
DM (g kg^−1^ of DM)	60	310.10	313.08	319.77	323.27	3.11	0.62
Crude protein (CP %)	60	3.88	3.90	3.45	3.28	0.50	0.12
NDF (g kg^−1^ of DM)	60	58.36	60.24	59.39	58.40	6.28	0.60
ADF (g kg^−1^ of DM)	60	38.15	39.83	38.98	38.26	4.67	0.46

a SP: control; SLP: *Lactobacillus plantarum*; SNP: natamycin; SLNP: *Lactobacillus plantarum* + natamycin.

## 4 Discussion

Nowadays, the most important problem in industry is preserving feed for a long time. Recently, different manufacturers have used different concentrations of natamycin to inhibit mold and yeast growth in their products. According to Mohamed *et al.*,^17^ natamycin at concentrations of 50 to 200 mg significantly affected the amount of fungal growth on media, dependent upon the experimental materials, environment, and the bacterial community. In our results, the different concentrations of natamycin had different effects on the growth of yeast, molds and bacteria, but the 0.5 g L^−1^, 1.00 g L^−1^, and 1.50 g L^−1^ concentrations of natamycin significantly (*p* < 0.05) reduced the growth of yeast, aerobic bacteria, and enterobacteria, while LAB was not influenced in fresh *Hybrid pennisetum* grass microbes and isolated microbes. Different concentrations of natamycin (5, 10 and 20 ppm) and potassium sorbate (0.5 and 1%) showed significant inhibitory effects on the growth of mold on Tallaga cheese after 30 days of storage at 4 ± 1 °C.^18^ This result is in agreement with Galal and Hameed,^19^ who ensiled feta cheese samples with different concentrations of natamycin (0.2% or 0.4%) for 2 months at 4 °C; when the cheese was analyzed, they could not detect the growth of molds or yeasts.

Some other antifungal chemical additives, such as formic acid, sodium benzoate, sodium propionate, potassium sorbate and propionic acid, are commonly used in the silage fermentation process. Suitable amounts of chemical supplementation decrease the silage pH and limit the loss of protein and carbohydrates throughout the ensiling process.^20^ Formic acid is commonly used in high-moisture silage (more than 70% moisture) for inhibiting Clostridial fermentation and protein degradation. With regards to animal performance, total dry matter intake and live weight gain were significantly increased with formic acid additive silage compared to control or other inoculant silages.^21^ Fresh corn after the inoculation of formic acid (98%), *L. plantarum* (3 × 10^10^ cfu g^−1^) and *Propionibacterium acidipropionici* (2.4 1/*t*), all treatments groups had lower pH as compared to the control, which decreased the *Clostridia* growth by lowering the levels of butyric acid, resulting in increased fermentation quality and inhibition of yeast and mold growth during 60 days of corn silaging.^22^

LAB and natamycin play an important role in the ensiling process. LAB compete with undesirable microorganisms for available (WSC) in the ensiling process. The combination of LAB with natamycin showed synergistic effects on fungal activity.^23^ Representatives of these harmful microorganisms are *Clostridia*, enterobacteria, and *Bacillus* spp. The major difference between the LAB and harmful bacteria is in the quality of the end products and the degradation of important nutrients. When lactic acid bacteria dominate, the silo pH value is ideally reduced to 4.6–4.1, reducing the yeast and mold growth over a period of several days and preserving the plant material.^24^ Natamycin also inhibits yeast, molds other undesirable microorganism and is the best feed or food preservation component. Yeast is the major microorganism to start aerobic degradation of silage, owing to its resemblance to glucose and lactate. Different concentrations of natamycin reduced the pH, WSC and yeast concentration while increasing the titratable acidity during different periods of yogurt storage.^25^

When using natamycin as an additive in yogurt at 10 and 20 mg kg^−1^, El-Diasty *et al.*^26^ also reported a decline of pH from 4.57 to 4.51, while the pH of the control decreased from 4.57 to 4.15 during 28 days of storage. In the present study, the aerobic bacteria (AB) and yeast were not affected at ensiling days 5 and 7, but they were significantly reduced by ensiling days 14, 30, and 60 (*p* > 0.05), while the lactic acid bacteria (LAB) were significantly (*p* < 0.05) influenced at all ensiling days and for all treatment groups, with the greatest significant (*p* > 0.05) influence seen in the SLP and SLNP groups. According to Balatsouras,^27^ the variation in the acidity level can also be recognized by the changes in the concentration of lactic acid. Mostly the additive materials increased the lactic acid levels during ensiling or storage, so there must be differences in the lactic acid concentrations between the natamycin and control fermentation groups. Hondrodimou *et al.*^3^ similarly reported the supplementation of natamycin in natural black olives. During the fermentation, the Enterobacteriaceae were enhanced hastily within the first 4 days, reaching the highest population of 4.9 log^10^ cfu mL^−1^. Yeasts coexisted mutually with lactic acid bacteria and followed a similar growth profile, and after the first 6 days of fermentation, the Enterobacteriaceae population declined to 2.1 log^10^ cfu mL^−1^. After 16 days, no Enterobacteriaceae were found in the natural black olive brine. At this time, the lactic acid bacteria population range was 5.58–5.89 log^10^ cfu mL^−1^. Pinto *et al.*^28^ reported that natamycin and *L. buchneri* deceased the pH and inhibited the yeast growth during and after the ensiling of maize silage. The supplementation of lactic acid bacteria (*L. plantarum*)^4,26^ and natamycin decreased the pH and increased the concentration of organic acid in the treatment groups as compared to the control. This was because the *L. plantarum* converted the WSC and secreted lactic acid and natamycin inhibited the growth of yeast and molds. Some other antifungal chemical reagents, such as formic acid, propionic acid, and sodium propionate, were used by Wang *et al.*^29^ as additives in alfalfa–corn mixed silage. At the silo opening day, the silage fermentation quality was enhanced with a higher concentration of lactic acid and lower pH, acetic acid (AA), ammonia nitrogen (NH_3_–N), yeast and molds. The higher concentrations of NH_3_–N and AA are known to increase the silage pH value, *Clostridia* and enterobacteria growth during the ensiling, which increases the butyric acid concentration and decreases the lactic acid and LAB, resulting in spoiled or poor quality silage. The lower levels of butyric acid during the ensiling inhibit *Clostridia* and enterobacteria growth, leading to the best silage quality.^30,31^

Air exposure is a very important issue for farmers because silage provides vital nutrients for animal production. However, when the silo is exposed to air then the silage is spoiled, which decreases the nutritional value.^8^ Natamycin is a highly effective component for reducing the growth of yeasts and molds and *L. plantarum* convert water-soluble carbohydrates into lactic acid to improve the silage quality. Our results showed that after three days of air exposure, the water-soluble carbohydrates, ammonia nitrogen, lactic acid, and acetic acid were not influenced, while pH and aerobic bacteria were significantly (*p* < 0.05) enhanced and lactic acid bacteria were significantly (*p* > 0.05) reduced. After air exposure in maize silage, Pinto *et al.*^28,30^ reported that the silos with natamycin alone and combined with *L. buchneri* showed the lowest pH and yeast values on day 1, while on day 3 the values were numerically changed in the natamycin additive group and on day 5 the yeast and pH were significantly increased in the natamycin alone and control groups as compared to the combined natamycin and *L. buchneri* group. Some other chemical additives can also improve the aerobic stability of silage; Zhang *et al.*^32^ used propionic acid, *L. plantarum* and *L. brevis* to improve the aerobic stability. During seven days of air exposure for mulberry silage, the pH value and organic acid, yeast, and ammonia nitrogen concentrations were stable on days three. After five days the pH value and yeast, ammonia nitrogen, and organic acid concentrations were significantly affected. The combined additives inoculated silage was stable for more than six days in the presence of air exposure. These results showed the synergistic effect of using homofermentative lactic acid bacteria in inhibiting silage from spoilage.^33^ After air exposure, the temperature in a silo will increase, leading to the microbial oxidation of acid and water-soluble carbohydrate convert into CO_2_ and H_2_O, resulting in silage spoilage. Shah *et al.*^12^ and Sifeeldein *et al.*^34^ strangely supported our results; they reported that water-soluble carbohydrates were not affected while the pH, yeast and aerobic bacteria were significantly increased during air exposure, and the lactic acid level and lactic acid bacteria were not influenced up to four days of air exposure, but after the ninth day of air exposure the lactic acid level and lactic acid bacteria were significantly decreased in Napier grass (*Pennisetum purpureum*) and King grass in inoculated silage. Wang *et al.*^10^ reported that the inoculation of antifungal chemical additives (propionic acid, sodium propionate, and formic acid) alone or in combination with lactic acid bacteria (*L. plantarum*) decreased the pH level and ammonia nitrogen concentration, while increasing the WSC levels in inoculated corn silage as compared to the control of corn silage. As a result, the corn was stable for seven days of air exposure with the addition of lactic acid bacteria, propionic acid, sodium propionate, and formic acid.

## 5 Conclusion

It is concluded that different concentrations of natamycin (0.5 g L^−1^, 1.00 g L^−1^ and 1.5 g L^−1^) significantly reduced the undesirable microbial community population in *Hybrid pennisetum* silage. The addition of *L. plantarum* CICC 20765 (1 × 10^5^ cfu g^−1^) in combination with natamycin (0.50 g L^−1^) reduced the levels of AA, pH, NH_3_–N, BA, and undesirable microbial community, and enhanced the chemical composition, fermentation quality, and air exposure. Natamycin alone did not significantly enhance the organic acid profile but improved the air exposure. More effort is needed to evaluate the effects on silage preservation on a large level and the effects on animal performance.

## Abbreviations

ADF: Acid detergent fiber
CP: Crude protein
DM: Dry matter
FW: Fresh weight
GFP: Green fluorescent protein
LAB: Lactic acid bacteria
NH_3_–N: Ammonia nitrogen
NDF: Neutral detergent fiber
SP: (Control)
SLP: *Lactobacillus plantarum*
SNP: Natamycin
SLNP: *Lactobacillus plantarum* + natamycin
WSC: Water-soluble carbohydrates

## Author contributions

A. A. Shah and X. Zhong conceived and designed the experiments; A. A. Shah, X. Zhang, Z. Tao, and Z. Liu , performed the experiments; Q. Chen, S. Khan, I. U. Khan and J. Wu, analyzed the data computationally; A. A. Shah, and X. Zhong wrote the manuscript.

## Funding

This work was supported by Jiangsu Agriculture Science and Technology Innovation Fund.

## Data availability statement

Not applicable. There is no supporting data.

## Ethics approval and consent to participate

Not applicable.

## Consent for publication

Not applicable.

## Conflicts of interest

The authors confirm that there are no conflicts of interest.

## Supplementary Material
